# Analysis of the SARS-CoV-2 spike protein glycan shield reveals implications for immune recognition

**DOI:** 10.1038/s41598-020-71748-7

**Published:** 2020-09-14

**Authors:** Oliver C. Grant, David Montgomery, Keigo Ito, Robert J. Woods

**Affiliations:** Complex Carbohydrate Research Center, University of Georgia, 315 Riverbend Rd, Athens, GA 30602 USA

**Keywords:** Immunology, Protein structure predictions

## Abstract

Here we have generated 3D structures of glycoforms of the spike (S) glycoprotein from SARS-CoV-2, based on reported 3D structures and glycomics data for the protein produced in HEK293 cells. We also analyze structures for glycoforms representing those present in the nascent glycoproteins (prior to enzymatic modifications in the Golgi), as well as those that are commonly observed on antigens present in other viruses. These models were subjected to molecular dynamics (MD) simulation to determine the extent to which glycan microheterogeneity impacts the antigenicity of the S glycoprotein. Lastly, we have identified peptides in the S glycoprotein that are likely to be presented in human leukocyte antigen (HLA) complexes, and discuss the role of S protein glycosylation in potentially modulating the innate and adaptive immune response to the SARS-CoV-2 virus or to a related vaccine. The 3D structures show that the protein surface is extensively shielded from antibody recognition by glycans, with the notable exception of the ACE2 receptor binding domain, and also that the degree of shielding is largely insensitive to the specific glycoform. Despite the relatively modest contribution of the glycans to the total molecular weight of the S trimer (17% for the HEK293 glycoform) they shield approximately 40% of the protein surface.

## Introduction

The current COVID-19 pandemic has led to over 11 million confirmed infections globally with a fatality rate of approximately 4.5%^1^ since the first reports of a severe acute respiratory syndrome (SARS) infection by a novel coronavirus (SARS-CoV-2) at the end of 2019. As of July 2020, there is still no vaccine or approved therapeutic to treat this disease. Here we examine the structure of the SARS-CoV-2 envelope spike (S) protein that mediates host cell infection, with a specific focus on the extent to which glycosylation masks this virus antigen from the host immune response.

Viral envelope proteins are often modified by the attachment of complex glycans that can account for up to half of the molecular weight of these glycoproteins, as in HIV gp120^2^. The glycosylation of these surface antigens helps the pathogen evade recognition by the host immune system by cloaking the protein surface from detection by the humoral and cellular components of the innate immune system^3–5^, and by altering the ability of the host to raise an effective adaptive immune response^6,7^ or even by enhancing infectivity^8^. Additionally, because the virus hijacks the host cellular machinery for replication and subsequent glycosylation, the viral glycan shield may be composed of familiar host glycans; thereby suppressing an anti-carbohydrate immune response^9^.

Fortunately, the innate immune system has evolved a range of strategies for responding to glycosylated pathogens^10^, but antigen glycosylation nevertheless complicates the development of vaccines^11^. Over time, the protein sequences in viral antigens undergo mutations (antigenic drift), which can alter the species specificity of the virus^12^, modulate its infectivity^13^, and alter the antigenicity of the surface proteins^14^. These mutations can also impact the degree to which the protein is glycosylated by creating new or removing existing locations of the glycans (glycosites) on the surface antigens^15,16^. Varying antigen glycosylation is thus a mechanism by which new virus strains can evade the host immune response^15^, and attenuate the efficacy of existing vaccines^11^.

Recently, a cryo-EM structure of the SARS-CoV-2 S glycoprotein has been reported^17^, which led to conclusion that, like the related protein from the 2002–2003 SARS pandemic (SARS-CoV-1)^18^, the CoV-2 S protein is also extensively glycosylated^17^. Furthermore, an analysis of the glycan structures present at each glycosite in the S trimer produced recombinantly in human embryonic kidney 293 (HEK293) cells has also been recently reported^19^.

The impact of glycosylation on the ability of antibodies to bind to a pathogenic glycoprotein may be estimated by quantifying the fraction of the surface area of the protein antigen that is physically shielded by glycans from antibody recognition. However, in contrast to proteins, glycans display large internal motions that prevents their accurate description by any single 3D shape^20,21^. Fortunately, MD simulations allow accurate prediction of the 3D shapes and motions of glycans, as confirmed by comparison to solution NMR data^22–24^, and such simulations have been widely applied to glycoproteins^25–29^.

Here we have generated 3D structures of several glycoforms of the SARS-CoV-2 S trimer glycoprotein, in which the glycans represent those present in the S protein produced in HEK293 cells^19^, as well as those corresponding to the nascent glycoprotein (prior to processing in the Golgi apparatus), as well as those that are commonly observed on antigens present in other viruses^28,30,31^. We have subjected these models to long replicate explicitly solvated molecular dynamics (MD) simulations and compared the extent to which glycan microheterogeneity impacts protein epitope exposure. The simulations were performed with the GLYCAM06/AMBER force field, which was developed for modeling carbohydrates, carbohydrate-protein complexes and glycoproteins^32–34^, and we use the data to assess the impact of glycosylation on the immunogenic and antigenic properties of the S glycoprotein. Additionally, we have identified peptides in the S protein that are likely to be presented in human leukocyte antigen (HLA) complexes, and discuss the role of S protein glycosylation in modulating the adaptive immune response to the SARS-CoV-2 virus or to a related vaccine.

## Results

### Model glycoforms

It is well established that there is a strong dependence of both the composition and relative glycan abundance (glycan microheterogeneity) on the cell type used for glycoprotein production. And there is a large body of data relating to the influence of host cell line on viral envelop protein glycosylation. For example, a glycomics analysis of influenza A virus produced in five different cell lines, all of relevance to vaccine production, led to the observation of profound differences in the compositions of the glycans at a given glycosite; with structures varying from paucimannose (Sf9 cells) to core-fucosylated hybrid with bisecting N-acetylglucosamine (Egg) to sialylated biantennary glycans (HEK293)^31^. For these reasons, we have generated five model glycoforms for the S glycoprotein: with reported site-specific glycosylation (HEK293)^19^, and as hypothetical homogeneously glycosylated glycoforms of the high mannose (M9), paucimannose (M3), biantennary complex (Complex) and core-fucosylated biantennary complex (Complex Core F) types. Comparisons between the glycoforms permits an assessment of the impact of differential levels of glycan processing on S protein antigenicity.

### Assessment of the impact of glycosylation on antigenicity

**Figure 1:**
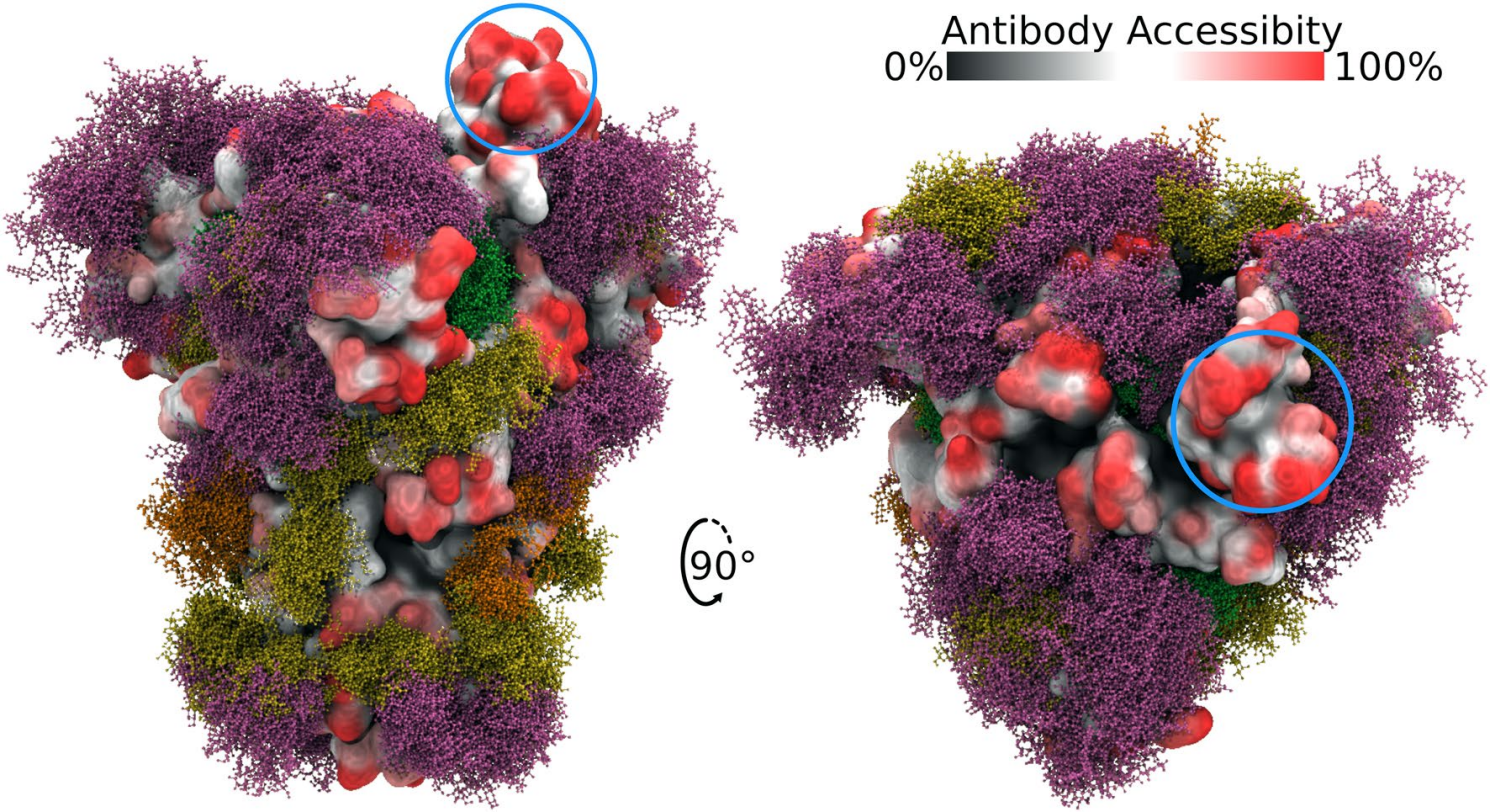
Side and top views of the S glycoprotein trimer with site-specific glycosylation shown as overlaid snapshots (moss surface) from MD simulations. The glycans are shown in ball-and-stick representation: M9 (green), M5 (dark yellow), hybrid (orange), complex (pink) (See Supplementary Table S1 for details). The protein surface is colored according to antibody accessibility from black to red (least to most accessible). The residues comprising the RBD in the “up” or “open” protomer are circled in blue. Images generated using Visual Molecular Dynamics (VMD)^35^ version 1.9.3.

We subjected the five glycoforms of the CoV-2 S glycoprotein to MD simulation and interpret the results in terms of the impact of glycan structure on the theoretical antigenic surface area of the S glycoprotein trimer (Fig. 1, Supplementary Figure S1, Table 1). A series of 3D structure snapshots of the simulation were taken at 1 ns intervals and analysed in terms of their ability to interact with a spherical probe based on the average size of hypervariable loops present in an antibody complementarity determining region (CDR) (see “Methods”). The surface accessibility of each amino acid in the trimer to the antibody probe was computed over the course of the simulations as the average antibody accessible surface area (AbASA). The per-residue AbASA values were plotted onto both the trimer 3D structure (Fig. 1) and sequence (Fig. 4), and the aggregate AbASA values reported for each glycoform in Table 1.

**Table 1:** SARS-CoV-2 S glycoprotein antigenic surface areas (Å^2^) as a function of glycoform.

Glycoform	Average antibody accessible surface area (AbASA)^a^	Percentage of trimer surface shielded from antibody recognition by glycans^b^	Glycan percentage of total molecular weight
	58,492 ± 2.2%	31 ± 0.7	11.7
	47,578 ± 3.7%	44 ± 1.6	21.6
	47,824 ± 4.8%	43 ± 2.1	19.4
	46,407 ± 2.5%	45 ± 1.1	20.7
HEK293 site-specific glycosylation	50,262 ± 2.6%	40 ± 1.0	17.0
Nude (non-glycosylated)	84,255 ± 2.2%	0	0

^a^Å^2^, computed with the naccess software^38^, version 2.1.1.

^b^(1 − AbASA/84,255)*100%.

The data indicate that uniform glycosylation with the smallest of the glycans (paucimannose, M3), which is a sub-structure common to all *N*-linked glycans, provided the least shielding of the trimer (31% coverage), leaving 69% of the surface exposed to an antibody probe relative to the same protein with no glycosylation. In contrast, the largest high mannose *N*-linked glycans (M9), which corresponds to the nascent glycoform that would exist prior to processing through the Golgi apparatus, led to a higher level of surface shielding (44%). The degree of cloaking offered by the two types of complex glycans are not significantly different from that of M9 at 43–45%. Glycosylation based on the HEK293 glycoform model resulted in 40% of the surface being shielded from antibody recognition. Unlike the extremely high level of glycan shielding in gp120 that hampers HIV vaccine development^36,37^, the level of shielding by glycans in the S protein is more moderate, with approximately 60% of the surface potentially accessible to antibodies.

The results from the AbASA analysis suggest that the overall antigenicity of the S protein is largely insensitive to glycan microheterogeneity, with the exception of the glycoform composed solely of M3 glycans. Nevertheless, differences in glycan microheterogeneity would be expected to modulate the binding to lectins associated with innate immune response and may impact the exposure of specific protein epitopes. Additionally, variations in glycan structure may affect local structural fluctuations in either the protein or glycan conformations, a feature that is poorly captured by the surface accessibility analysis.

A visual examination of the glycoform 3D structures (Fig. 1 and Supplementary Figure S1) indicates that the most exposed protein epitopes comprise the ACE2 receptor site, specifically the apex region of the S1 domain when that domain is in the “up” or “open” conformation. It can also be observed that a ring of antigenic sites appears to encircle the S1 domain, independent of glycoform. To corroborate these theoretical predictions we then analysed the experimentally reported epitopes for related S trimer–monoclonal antibody (mAb) interactions.

### Comparison with epitopes in related coronavirus S glycoproteins

**Figure 2:**
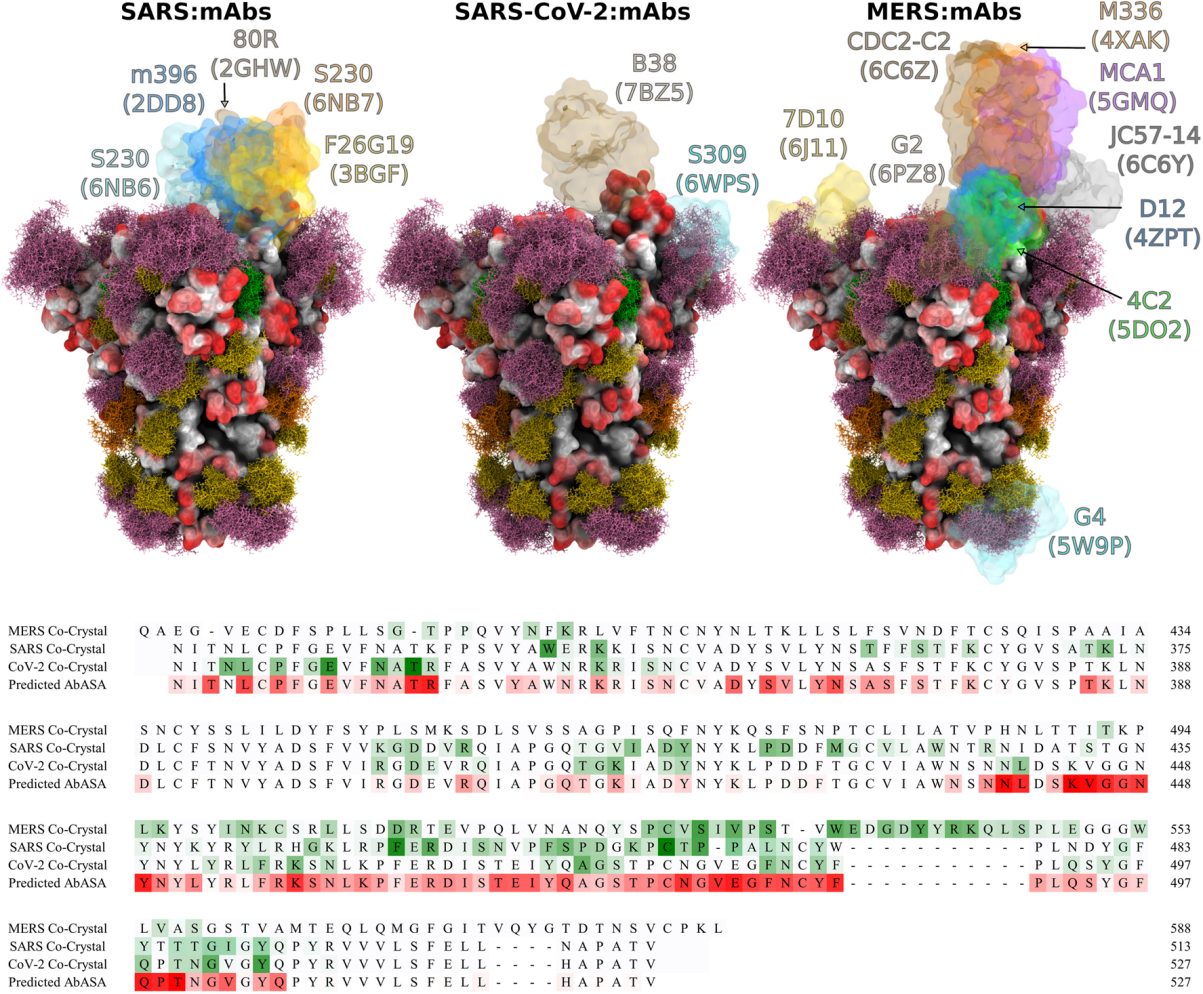
Superimpositions of neutralizing antibodies from co-complexes for the SARS, MERS and SARS-CoV-2 S proteins onto the HEK293 S trimer model for SARS-Cov-2. Upper panels, the antibody fragments are shown as pastel transparent surfaces with the mAb name and PDB ID for each co-complex shown in the same color^40–52^. Lower panel, the alignments of the RBD sequences of MERS, SARS, and CoV-2 spike proteins^53^ with the experimentally derived antibody contact areas shaded from white to green (least to most contact) compared to the predicted AbASA values for the HEK293 glycoform, shaded from white to red (least to most exposed). Glycosites in the SARS-CoV-2 sequence are indicated with an asterisk above the aligned sequences. Images generated using VMD^35^ version 1.9.3; antibody contact areas computed with the naccess software^38^ using a water-sized probe.

To illustrate the location of known epitopes and to assess the impact of glycosylation on epitope exposure, we aligned the 3D structures of the spike proteins from SARS-CoV-2 (CoV-2) with those from co-crystal structures of SARS-CoV-1 (SARS) and Middle East Respiratory Syndrome CoV (MERS) that contained bound mAb fragments. The S trimers of SARS and CoV-2 share a high degree of structural similarity, with an average root-mean-squared difference (RMSD) in the Cα positions of only 3.8 Å^17^. The MERS S glycoprotein also shares a similar trimeric structure with SARS and CoV-2. From this alignment, the preference of neutralizing antibodies to bind to the RBD in these coronaviruses is apparent. The extent to which epitopes in the CoV-2 S trimer might be inaccessible to known antibodies on the basis of structural differences in the RBD orientations or due to shielding by glycans on the CoV-2 S trimer surface may also be inferred (Fig. 2).

Of the S glycoprotein residues observed to interact with neutralizing CoV-2 antibodies, 94% were predicted to be accessible to antibody binding from the AbASA analysis of the HEK293 glycoform (Fig. 2 and Supplementary Figure S3). With the exception of mAb G4 (PDB ID 5W9P), these epitopes are all in or proximal to the RBD, providing a structural rationale for the neutralizing ability of these mAbs. However, the AbASA analysis also predicted a significant number of epitopes distal to the RBD for which crystallographic antibody co-complexes have not yet been reported. This may reflect the fact that neutralizing antibodies are most often the focus of such structural studies. It is worth noting that a recent screening of serum from confirmed COVID-19 patients^39^ confirmed the presence of additional immunodominant linear epitopes that are not in the RBD, as predicted by the AbASA analysis to be antigenic sequences (Supplementary Figure S3).

**Figure 3:**
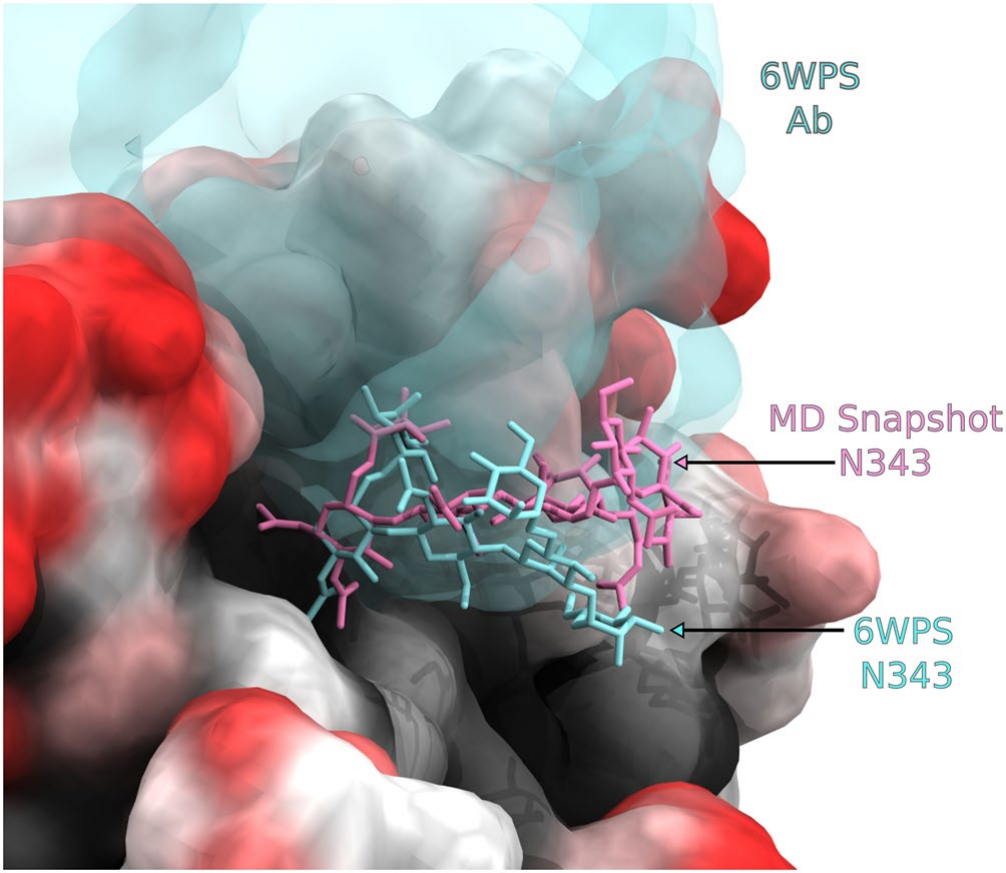
Image of the S309 antibody (cyan) observed in the crystallographic co-complex (PDB ID 6WPS^54^) compared to a single pose from an MD simulation of the S-glycoprotein trimer. While numerous poses of the glycan at N343 were incompatible with antibody binding, there are poses within the MD trajectory that are similar to that found in the crystal structure co-complex with S309 that would permit binding.

The antibody contact data in Fig. 2 show a remarkable degree of epitope conservation among each of the coronaviruses, and a strong correlation between the predicted S glycoprotein antibody accessibility and the observed antibody epitopes. However, a closer examination also indicates a contraction between the 3D glycoform model and the observed binding of the neutralizing antibody S309 (PDB ID 6WPS). The moss plot (Fig. 2) of the glycan at N343 (sequence VFNATR) indicates that there would be a high degree of overlap expected between the glycan and mAb S309. Despite this visual inference, the quantitative AbASA accessibility analysis is in good agreement with the experimental epitope contact areas. While the moss plot representation provides a clear indication of the extent of glycan motion and illustrates that no single static model could fully capture the degree of glycan shielding, it can also overemphasize the extent to which a glycan might cloak the protein surface from mAb recognition. While many glycan poses observed during the MD simulation were incompatible with mAb S309 binding, a subset of poses could be identified in the MD trajectory that would permit the binding of this mAb (Fig. 3). Thus, the degree of shielding is better captured by the more detailed AbASA analysis rather than by a visual overlay of structures.

### Adaptive immune response to SARS-CoV-2

**Figure 4:**
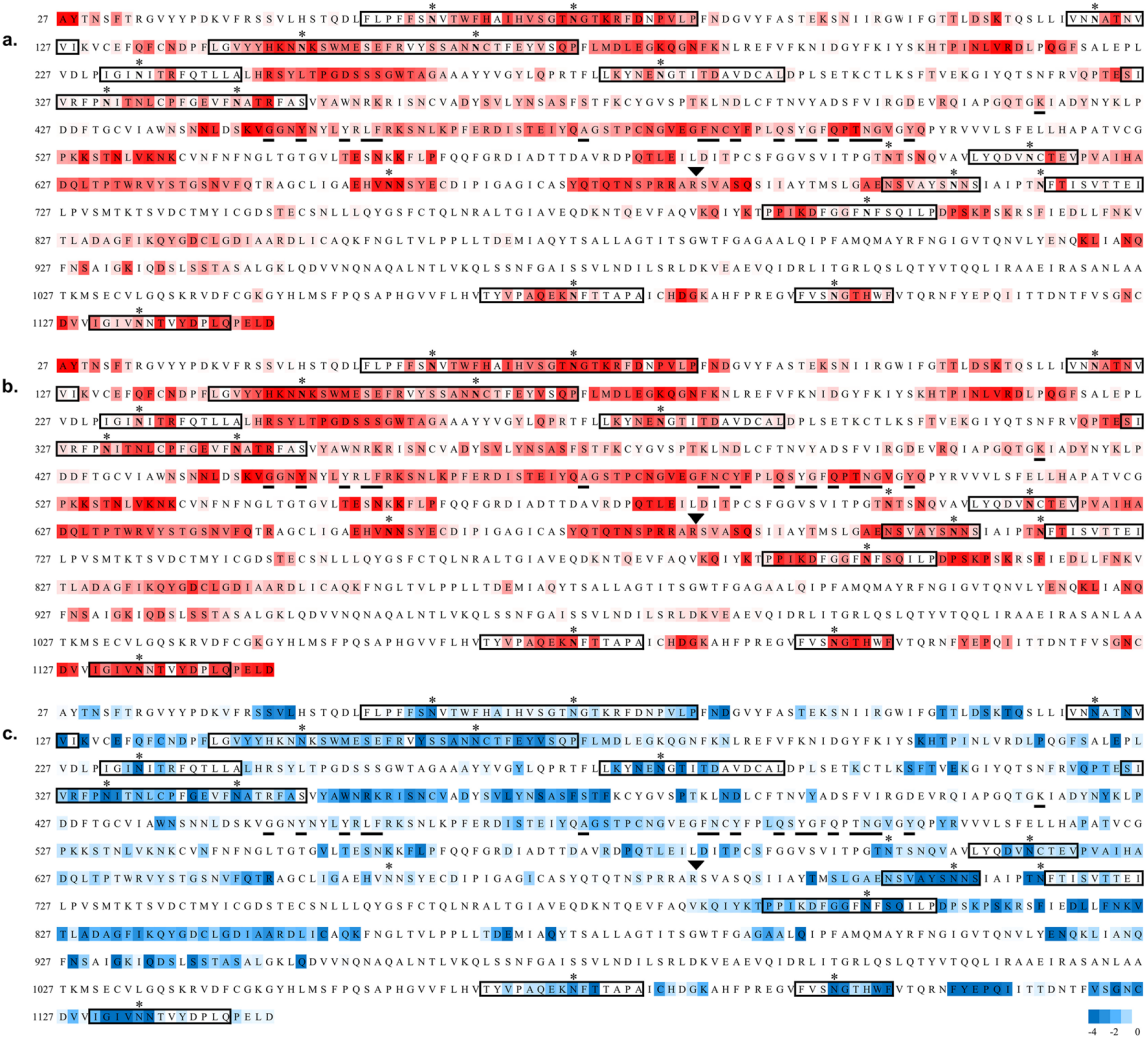
Sequence of the S protein (NCBI: YP_009724390.1) used to generate the 3D model of the glycoprotein. Residues 1–26 and 1,147–1,273 were not included in the 3D structure due to a lack of relevant template structures. Sequences within a rectangle were predicted to consist of one or more HLA antigens using the RankPep server (imed.med.ucm.es/Tools/rankpep^55,56^). Glycosites are indicated with asterisks, residues reported to interact with the ACE2 receptor^62^ are underlined, and the protease cleavage site is indicated with a triangle above the RS junction. (**a**) The sequence is colored according to antibody accessibility computed for the site-specific glycoform from white to red (least to most accessible). (**b**) Antibody accessibility computed for the non-glycosylated (nude) protein. (**c**) The difference in accessibilities between the site-specific and non-glycosylated glycoforms is plotted as the fold change in epitope accessibility during the simulation, from − 4 (blue) to 0 (white), where blue indicates glycosylation-dependent surface shielding.

Beyond a role in shielding the underlying protein from recognition by antibodies, the glycans on pathogenic proteins may also attenuate the ability of the host immune system to raise antibodies against any epitopes that include the glycan. In a T-cell dependent adaptive immune response, peptides from the pathogen are presented on antigen presenting cells by major histocompatibility complex II molecules, also known as human leukocyte antigen (HLA) complexes. HLA complexes have preferred peptide antigen motifs, and based on a knowledge of these preferences it is possible to predict which peptides in a pathogenic protein are likely to be HLA antigens^55,56^. However, when that peptide contains a glycosylation site, the ability of the peptide to be presented in an HLA complex may be compromised, if for example the peptide cannot bind to the HLA molecule due to the steric presence of the glycan. However, glycopeptides may be presented in HLA complexes if the glycan is small enough^57^ or if it is found on the end of the peptide antigen where it doesn’t interfere with HLA binding^58^. The glycan-mediated shielding of predicted HLA antigens (Supplementary Table S2) derived from the S glycoprotein are shown in Fig. 4 and Supplementary Figures S2 and S4 for all HLA peptide sequences that also contain a glycosite.

As expected, glycosylation consistently decreased the surface exposure of the residues proximal to the glycosites (Fig. 4c), but also led to non-sequential changes in exposure, as a result of the 3D topology of the protein surface in the vicinity of each glycosite. Of the 18 glycosites in the 3D structure, 16 are present in putative HLA peptides. Although the glycans may occur throughout the HLA sequences (Supplementary Table S2), in 12 of these sequences the glycans are predicted to be present at the terminus of at least one putative HLA antigen. This observation suggests that these 12 glycosites may not interfere with antigen presentation in an HLA complex. This property is essential for the potential generation of antibodies against the underlying epitopes, and may lead to antibodies that target these carbohydrates on the S glycoprotein^57^. As a case in point, glycosite N343 is predicted to exist in an immunogenic HLA sequence (Supplementary Table S2), and recently a co-crystal structure of the SARS-CoV-2 S trimer has been reported in which a neutralizing antibody (S309) interacts with this epitope and with the glycan at N343^54^. Anti-carbohydrate antibodies have been shown to be neutralizing in other viruses, such as HIV^59^, and therefore glycosylated peptides can offer an alternative to more traditional peptide epitopes. Glycan immunogenicity is enhanced when the glycans or their clusters are significantly different from self, and thus are less immunologically tolerated^9^. Although viruses exploit the host glycosylation machinery in their biosynthesis, differences from typical host glycan distributions can occur when for example the virus cloaks itself so densely in glycosites that the glycans are not accessible to glycan processing enzymes, due to steric crowding, and remain in their high mannose form^28^. Examples of this are seen in the high-mannose clusters in some strains of influenza^28^ and in HIV^60^. From the perspective of vaccine development^61^, targeting glycans as epitopes would be expected to benefit from matching the glycan microheterogeneity in the vaccine to that in the circulating virus, which requires appropriate consideration of the choice of cell type for vaccine production^31^.

## Discussion

The present study indicates that glycans shield approximately 40% of the underlying protein surface of the S glycoprotein trimer from antibody recognition, and that this value is relatively insensitive to glycan type. This suggests that although viral glycan microheterogeneity varies according to host cell type and potentially therefore between infected individuals, the efficacy of antisera should not be profoundly impacted by such differences. This conclusion is consistent with emerging data that indicate the efficacy of convalescent sera^63–65^ in treating COVID-19 patients.

The observation that homogeneously glycosylated glycoforms are predicted to display approximately the same level of antibody shielding as those computed for the more relevant site-specific glycoform suggests that hypothetical models of glycosylation can be usefully applied in advance of the report of experimental glycomics data. As in all MD simulations, accurate and adequate sampling of the conformational poses of the glycans and the protein is essential to avoid anecdotal results. Here, multiple independent simulations were performed into the μs regime. The ability to predict the impact of protein glycosylation on epitope recognition is significant as it enables the effects of glycosite alterations to be estimated in anticipation of seasonal antigenic drift. By analogy with influenza^66,67^, variations in glycosite location arising from antigenic drift can be expected to have a profound effect on SARS-CoV-2 S protein antigenicity and potentially vaccine efficacy. Fortunately, the most accessible and largest epitope in the S trimer consists of the ACE2 binding domain, where the virus cannot exploit glycan shielding or mutational changes to evade host immune response without potentially attenuating its fitness. The requirement that the virus maintain the integrity of the ACE2 RBD suggests that a vaccine that includes this epitope may maintain efficacy despite antigenic drift, as long as the virus continues to target the same host receptor.

While overall shielding of the underlying protein surface does not appear to be highly sensitive to glycan microheterogeneity, it could certainly impact the innate immune response by altering the ability of collectins and other lectins of the immune system to effectively bind to the S glycoprotein and neutralize the virus^28^, and may impact the adaptive immune response by altering the number of viable HLA antigens. Given that in humans, glycan microheterogeneity varies between individuals, and depends on many factors, including age^68,69^, underlying disease^70,71^ and ethnicity^72^, access to 3D models of the S glycoprotein may aid in defining the molecular basis for the observed differential susceptibilities among individuals to COVID-19^73,74^.

The level of agreement between the predicted epitopes based on MD simulation of the S trimer and those observed by epitope mapping or in co-crystal structures, suggests the AbASA approach to epitope identification could play a useful role in guiding the development of vaccine strategies as well as in interpreting differences in the responses of individuals to either the disease severity or vaccine efficacy.

## Methods

### SARS-CoV-2 spike (S) protein structure

A 3D structure of the prefusion form of the S protein (RefSeq: YP_009724390.1, UniProt: P0DTC2 SPIKE_SARS2), based on a Cryo-EM structure (PDB code 6VSB)^17^, was obtained from the SWISS-MODEL server (swissmodel.expasy.org). The model has 95% coverage (residues 27 to 1,146) of the S protein.

### S trimer glycoform generation

Five unique 3D models for the glycosylated glycoprotein were generated using the glycoprotein builder available at GLYCAM-Web (www.glycam.org) together with an in-house program that adjusts the asparagine side chain torsion angles and glycosidic linkages within known low-energy ranges^75^ to relieve any atomic overlaps with the core protein, as described previously^66,76^. The site specific glycans used to model a glycoform representative of the data obtained from the S glycoprotein expressed in HEK293 cells^77^, are presented in Supplementary Table S1.

### Energy minimization and molecular dynamics simulations

Each glycosylated structure was placed in a periodic box of approximately 130,000 TIP3P water molecules^78^ with a 10 Å buffer between the glycoprotein and the box edge. Energy minimization of all atoms was performed for 20,000 steps (10,000 steepest decent, followed by 10,000 conjugant gradient) under constant pressure (1 atm) and temperature (300 K) conditions. All MD simulations were performed under nPT conditions with the CUDA implementation of the PMEMD^79,80^ simulation code, as present in the Amber14 software suite^81^. The GLYCAM06j force field^82^ and Amber14SB force field^83^ were employed for the carbohydrate and protein moieties, respectively. A Berendsen barostat with a time constant of 1 ps was employed for pressure regulation, while a Langevin thermostat with a collision frequency of 2 ps^−1^ was employed for temperature regulation. A nonbonded interaction cut-off of 8 Å was employed. Long-range electrostatics were treated with the particle-mesh Ewald (PME) method^84^. Covalent bonds involving hydrogen were constrained with the SHAKE algorithm, allowing an integration time step of 2 fs^85^ to be employed. The energy minimized coordinates were equilibrated at 300 K over 400 ps with restraints on the solute heavy atoms. Each system was then equilibrated with restraints on the Cα atoms of the protein for 1 ns, prior to initiating 6 independent production MD simulations with no restraints applied for a total time of 3 μs for the HEK293 glycoform, or 3 runs for a total of 1 μs for the homogeneously glycosylated theoretical glycoforms.

### Antigenic surface analysis

**Figure 5:**
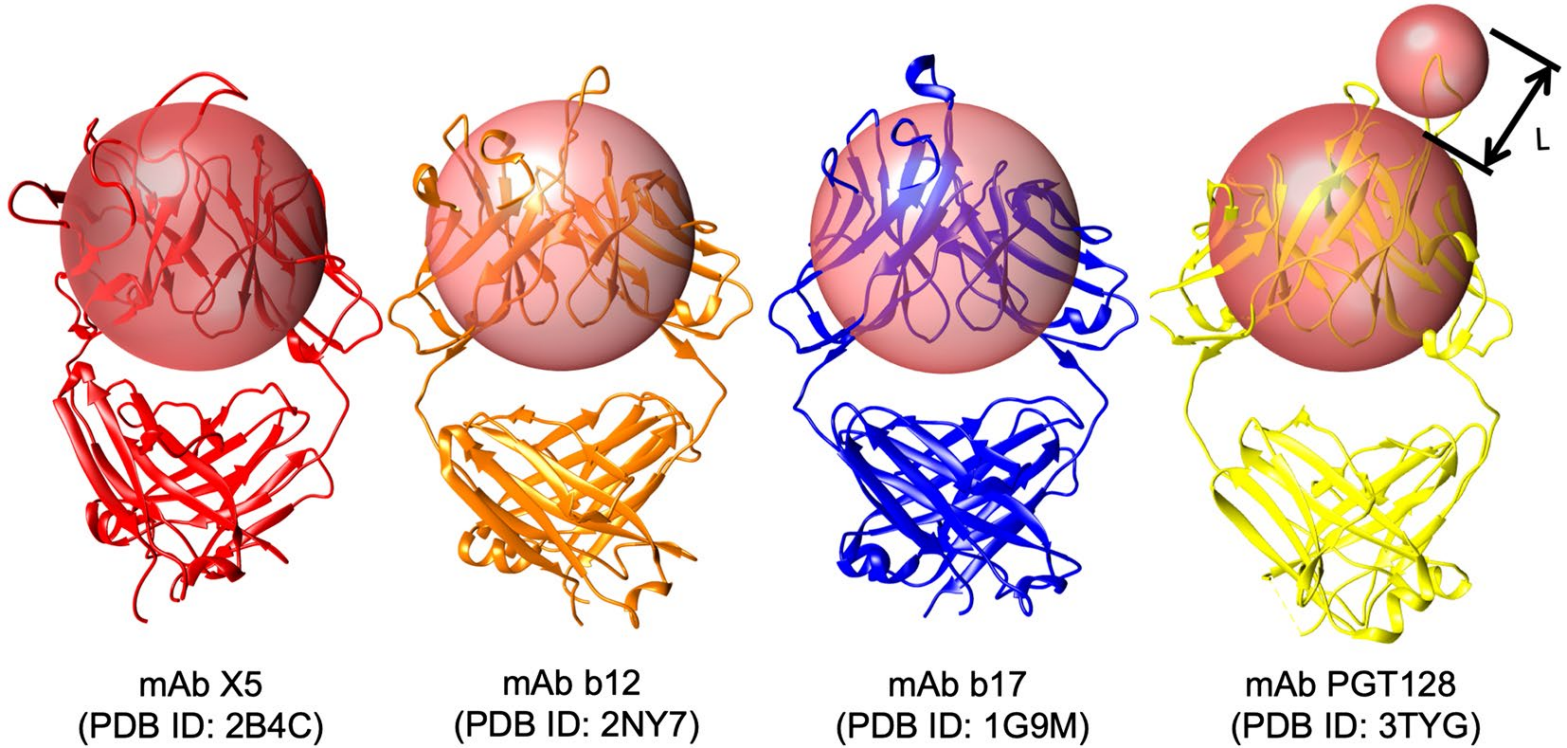
Antibody accessible surface area estimation using a pair of spherical probes. To estimate the AbASA, a CDR spherical probe was derived (radius 7.2 Å, smaller sphere) that approximates the average size of the hypervariable loops in the CDR from four anti-gp120 antibodies, in which the epitopes were either protein surface residues (PDB IDs: 2B4C^87^, 2NY7^88^, 1G9M^89^) or both carbohydrate and protein residues: (3TYG^90^). Additionally, to account for the presence of the beta-sheet framework in the antibody variable fragment (Fv), we introduced a second larger probe (18.6 Å) sufficient to approximately enclose that domain. Images generated with UCSF Chimera^91^.

The antigenic surface area was calculated as the sum of the surface areas of any protein residues that make contact with the CDR probe, provided that the CDR probe is proximal to the Fv probe (Fig. 5). This latter requirement is governed by “L”, which requires that the distance between the CDR-antigen contact site and the Fv probe surface be less than the length (10.4 Å) of the longest CDR loop in mAb PGT128. PGT128 was chosen for this reference as it contains a particularly long CDR loop that penetrates the glycan shield of gp120. These probe sizes may be compared to values of 5 and 10 Å employed previously to estimate antigenic surface area^86^. Changes in the solvent accessible surface area (SASA) showed no significant shielding by glycans and thus a simple SASA model (radius 1.4 Å) was not useful for this analysis^86^. Failure to include the Fv domain probe, that is, probing the surface only with the smaller 7.2 Å sphere, led to the detection of contacts in narrow deep crevasses on the protein surface (Supplementary Figure S5), that while theoretically possible, would require an exceptionally long hypervariable loop.

## Supplementary information

AMBER topology (TOP), trajectory (NETCDF), and coordinate (PDB) files for each glycoform of the S glycoprotein are available for download from GLYCAM-Web (https://www.glycam.org) and from figshare (https://doi.org/10.6084/m9.figshare.12273188.v2).
