# Supplementary material for: Analysis of the SARS-CoV-2 spike protein glycan shield reveals implications for immune recognition
Source: Sci Rep. 2020 Sep 14;10:14991. doi: 10.1038/s41598-020-71748-7 (PMC7490396; doi:10.1038/s41598-020-71748-7)
Supplement: Supplementary file 1 — AMBER topology (TOP), trajectory (NETCDF), and coordinate (PDB) files for each glycoform of the S glycoprotein are available for download from GLYCAM-Web (https://www.glycam.org) and from figshare (https://doi.org/10.6084/m9.figshare.12273188.v2). [file 41598_2020_71748_MOESM1_ESM.pdf]

Supplementary Information for:

Analysis of the SARS-CoV-2 spike protein glycan shield reveals implications for immune recognition

Oliver C. Grant, David Montgomery, Keigo Ito, Robert J. Woods*

Complex Carbohydrate Research Center, University of Georgia, 315 Riverbend Rd, Athens, GA 30602

Corresponding Author
*Mailing address: 315 Riverbend Road, Athens, GA 30602.
Tel.: 706-542-4454. Fax: 706-542-4412. E-mail: rwoods@ccrc.uga.edu

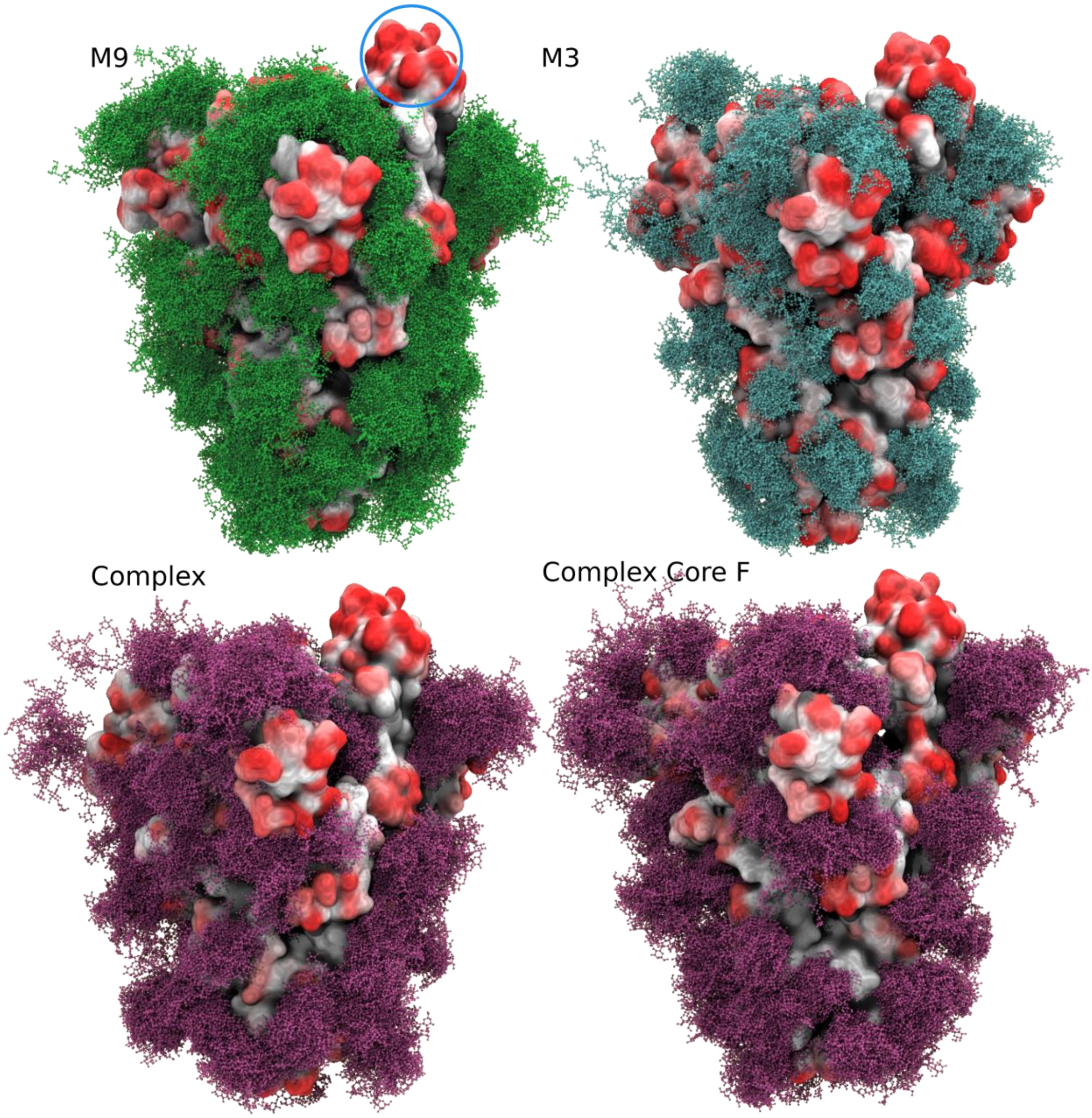

Supplementary Figure S1. Overlay of snapshots from MD simulation of the S glycoprotein with homogeneous glycosylation. The glycans are shown in ball-and-stick representation: M9 (green), M3 (cyan), complex and complex core F (pink) (See Table S1 for details). The protein surface is colored according to antibody accessibility from black to red (least to most accessible). Images generated using Visual Molecular Dynamics (VMD) [62] version 1.9.3.

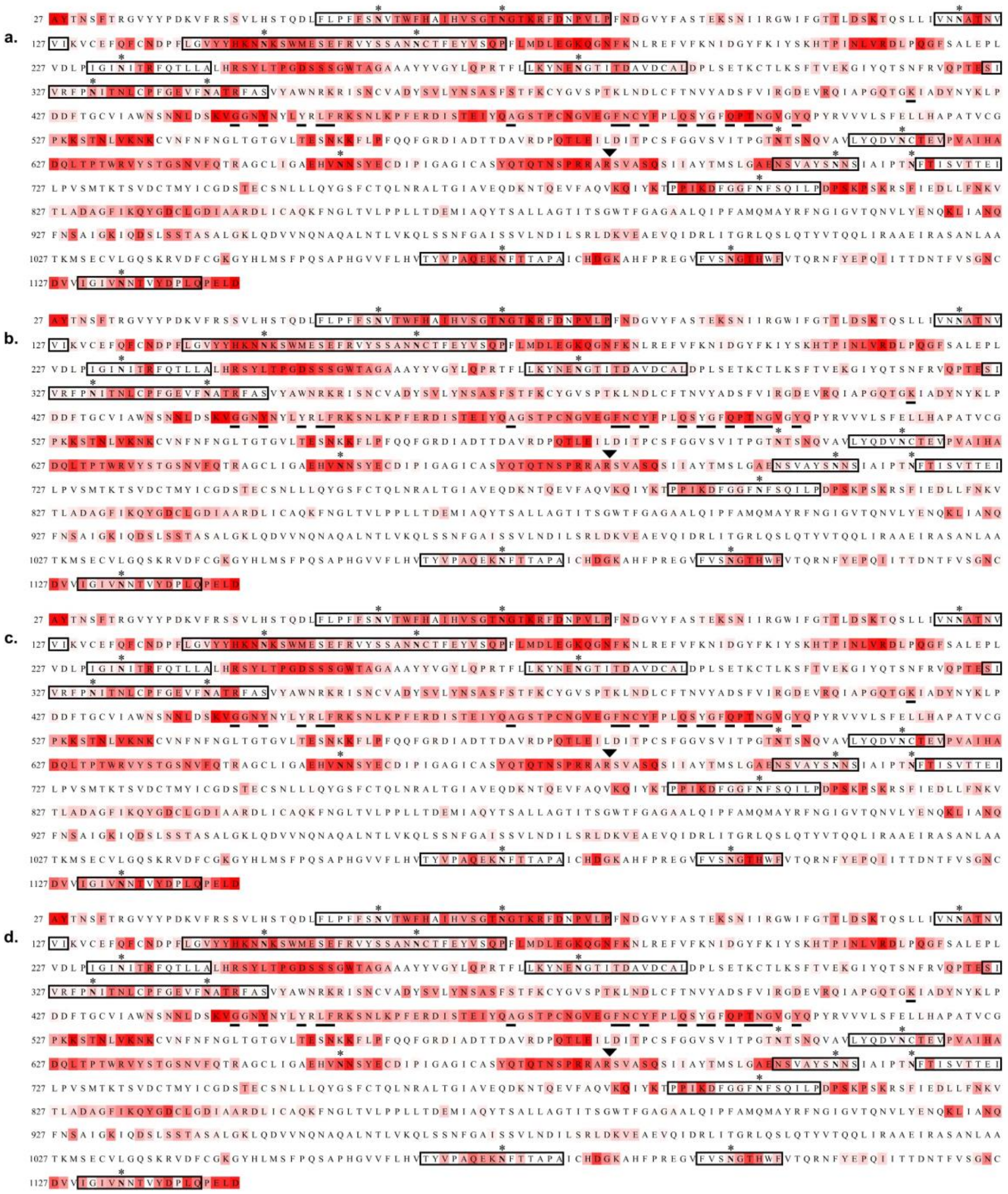

Supplementary Figure S2. Sequence of the S protein model colored by antibody accessibility from white to red (0 to 100 % accessible) computed for the M3 (**a.**), M9 (**b**.), Complex (**c.**), and Complex Core F (**d.**) glycoforms. Glycosites are indicated with asterisks, residues reported to interact with the ACE2 receptor are underlined, and the protease cleavage site is indicated with a triangle above the RS junction. Sequences within a rectangle were predicted to consist of one or more HLA antigens using the RankPep server (imed.med.ucm.es/Tools/rankpep[55,56]).

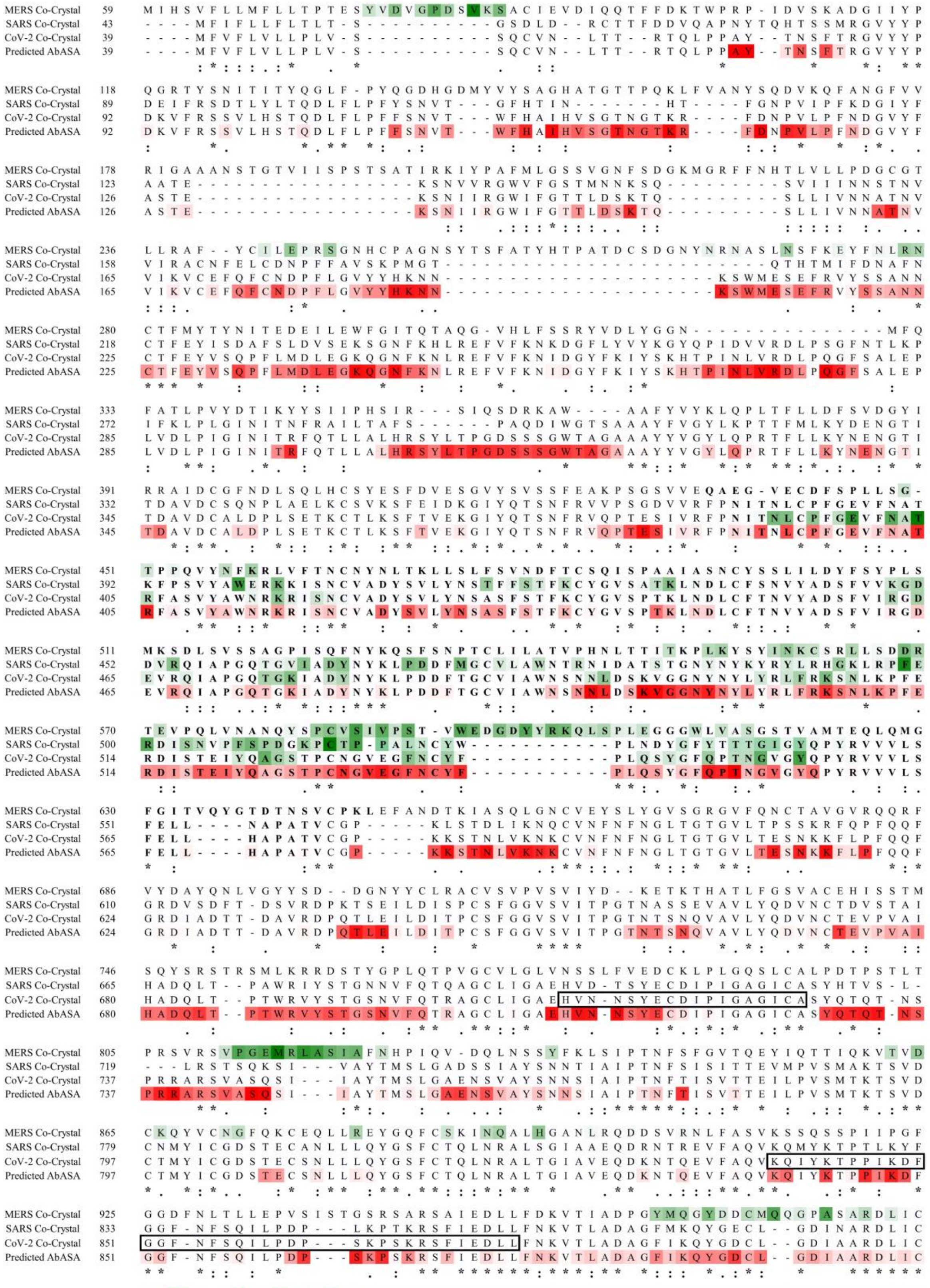
MERS Co-Crystal
SARS Co-Crystal
CoV-2 Co-Crystal
Predicted AbASA

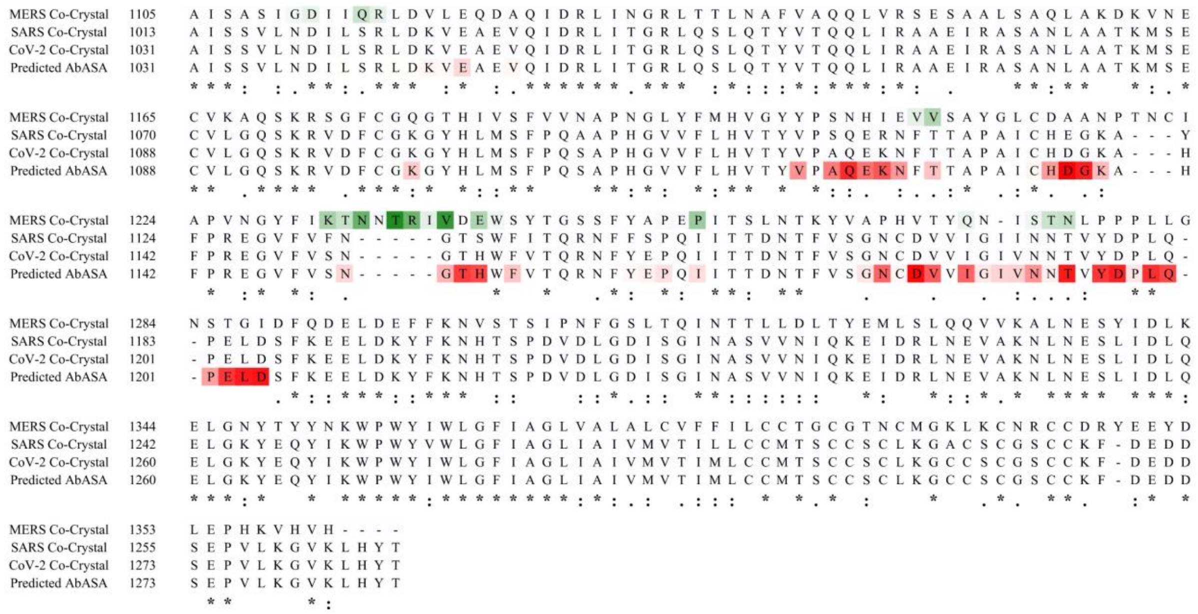

Supplementary Figure S3. Sequence alignment of MERS, SARS, and CoV-2 spike proteins. The MERS, SARS, and CoV-2 sequences are colored from white to green (no antibody shielding to highly shielded) based on residues interacting with the antibodies depicted in Figure 3. This is calculated by taking the difference in residue accessibility between the antibody co-complex and the spike protein without the antibody. The predicted AbASA data is colored from white (no accessibility) to red (high accessibility). Sequences within a rectangle were recently shown to be immunodominant epitopes[39], and the RBD is highlighted in bold.

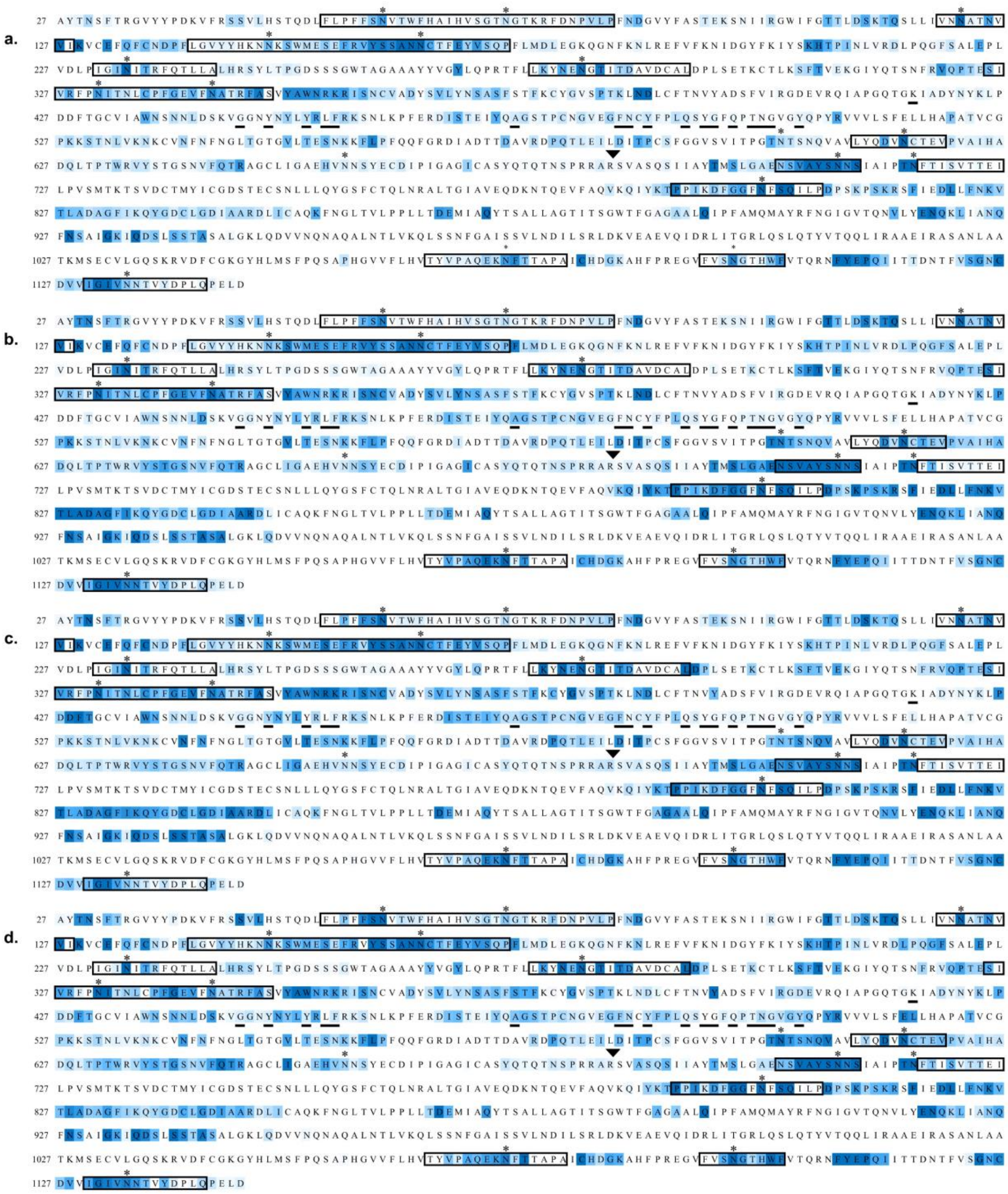

Supplementary Figure S4. Sequence of the S protein with the difference in antibody accessibilities between glycoforms plotted as the fold change in accessibility from -4 to 0 (blue to white), where blue indicates glycosylation-dependent surface shielding, computed for the M3 (**a.**), M9 (**b.**), Complex **(c.**), and Complex Core F (**d.**) glycoforms. Glycosites are indicated with asterisks, residues reported to interact with the ACE2 receptor are underlined, and the protease cleavage site is indicated with a triangle above the RS junction. Sequences within a rectangle were predicted to consist of one or more HLA antigens using the RankPep server (imed.med.ucm.es/Tools/rankpep[55,56]).

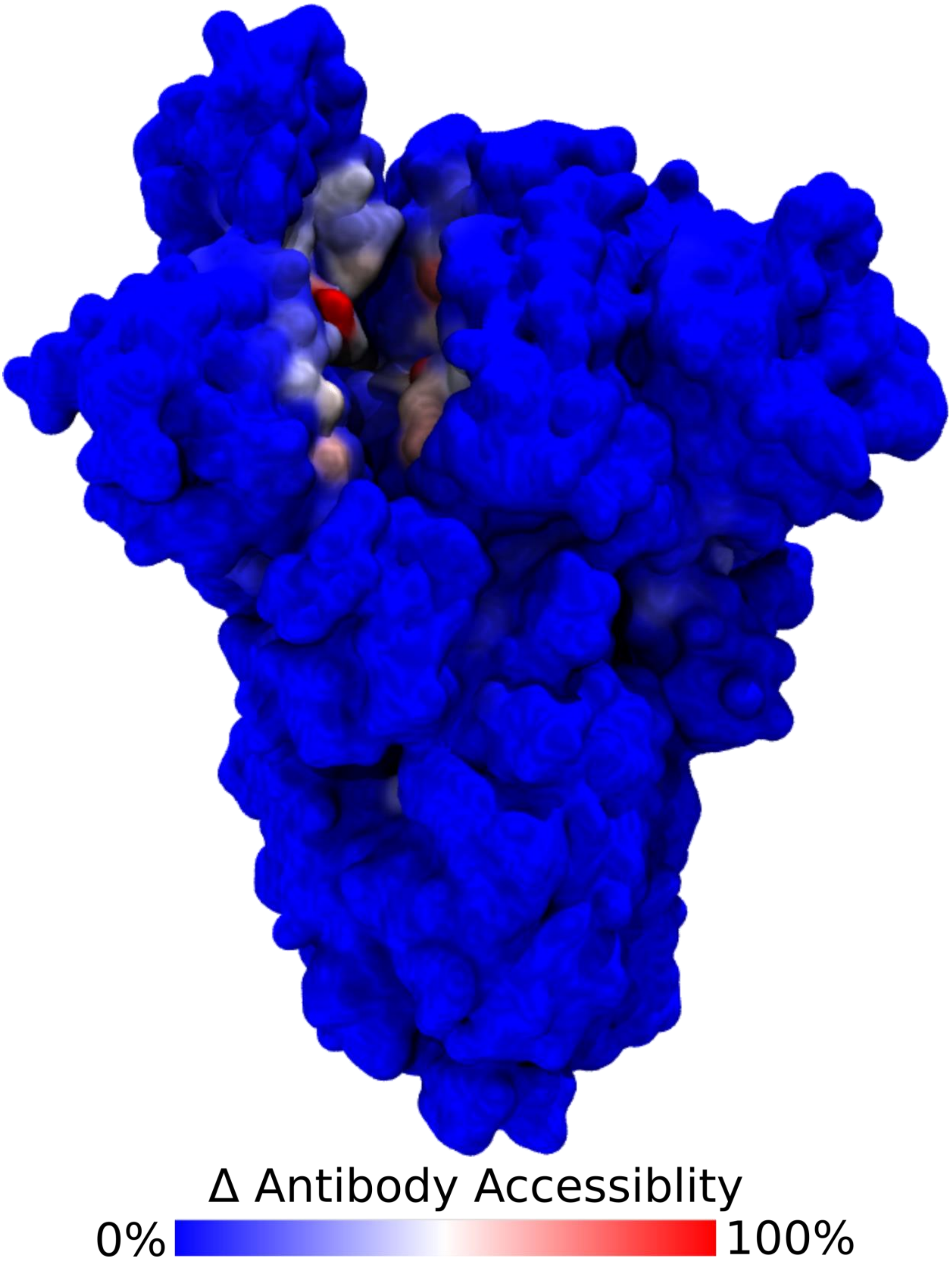

Supplementary Figure S5. The difference in AbASA calculations between using just the small probe, and the small probe in combination with the large probe. This difference is plotted onto the SARS-CoV-2 spike protein from blue (no difference) to red (100% difference).

Supplementary Table S1. Structures of the glycans attached at each glycosite in the Site Specific Model

Hybrid[a] 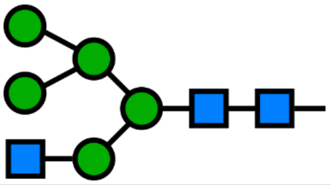	DManpα1-6[DManpα1-3]DManpα1-6[DGlcpNAcβ1-2DManpα1-3]DManpβ1-4DGlcpNAcβ1-4DGlcpNAcβ1-OH<br><br>Glycosite residue: 657
FA2 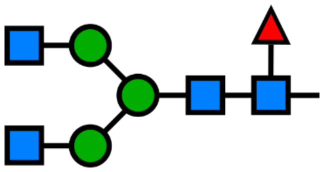	DGlcpNAcβ1-2DManpα1-6[DGlcpNAcβ1-2DManpα1-3]DManpβ1-4DGlcpNAcβ1-4[LFucpα1-6]DGlcpNAcβ1-OH<br><br>Glycosite residues: 149, 165, 331, 343, 616, 1134
A2 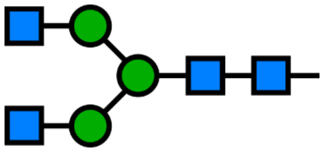	DGlcpNAcβ1-2DManpα1-6[DGlcpNAcβ1-2DManpα1-3]DManpβ1-4DGlcpNAcβ1-4DGlcpNAcβ1-OH<br><br>Glycosite residue: 1098
FA2B 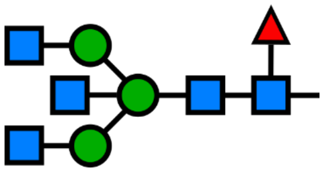	DGlcpNAcβ1-2DManpα1-6[DGlcpNAcβ1-4][DGlcpNAcβ1-2DManpα1-3]DManpβ1-4DGlcpNAcβ1-4[LFucpα1-6]DGlcpNAcβ1-OH<br><br>Glycosite residues: 74, 282
M5 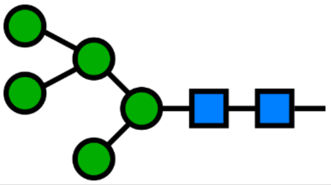	DManpα1-6[DManpα1-3]DManpα1-6[DManpα1-3]DManpβ1-4DGlcpNAcβ1-4DGlcpNAcβ1-OH<br><br>Glycosite residues: 61, 122, 603, 709, 717, 801, 1074
M8 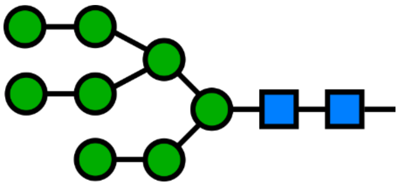	DManpα1-2DManpα1-6[DManpα1-2DManpα1-3]DManpα1-6[DManpα1-2DManpα1-3]DManpβ1-4DGlcpNAcβ1-4DGlcpNAcβ1-OH<br><br>Glycosite residue: 234

[a]Glycan naming system from reference 15. In cases where the mass spectrometry data allows for multiple structures, such as FA2/FA1B, the glycan with the least ambiguity was selected. For example, FA2 was selected over FA1B, as the position of the single antennae is ambiguous.

Supplementary Table S2. Peptides containing glycosites with sequences predicted[a] to bind to human HLAs

HLA Position Specific Scoring Matrix	RANK	SEQ. POS.	N	SEQUENCE	C	HLA Site	SCORE	% OPT.
HLA_DR15oDRB1s1501c	5	55	QDL	FLPFFS**NVT**	WFH	1	15.3	36.8
HLA_DR4	4	58	FLP	FFS**NVT**WFH	AIH	1	13.3	32.2
HLA_DR4oDRB1s0401c	3	58	FLP	FFS**NVT**WFH	AIH	1	17.6	40.0
HLA_DQ7oDQB1s0301c	13	61	FFS	**NVT**WFHAIH	VSG	1	11.7	25.7
HLA_DR8oDRB1s0801c	6	61	FFS	**NVT**WFHAIH	VSG	1	14.7	31.2
HLA_DR1	20	63	S**NV**	**T**WFHAIHVS	GTN	1	8.8	18.8
HLA_DR4	21	64	**NVT**	WFHAIHVSG	TNG	1	10.7	25.8
HLA_DR4oDRB1s0401c	10	64	**NVT**	WFHAIHVSG	TNG	1	12.1	27.5
HLA_DR15oDRB1s1501c	21	68	FHA	IHVSGT**NGT**	KRF	2	10.9	26.2
HLA_DR3	7	75	GT**N**	**GT**KRFDNPV	LPF	2	11.7	29.0
HLA_DR1	18	76	T**NG**	**T**KRFDNPVL	PFN	2	9.3	19.7
HLA_DR15oDRB1s1501c	14	77	**NGT**	KRFDNPVLP	FND	2	12.8	30.7
HLA_DR15oDRB1s1501c	7	120	LLI	VN**NAT**NVVI	KVC	3	14.9	35.6
HLA_DR8oDRB1s0801c	2	141	DPF	LGVYYHKN**N**	**KS**W	4	17.1	36.2
HLA_DR8oDRB1s0801c	1	142	PFL	GVYYHKN**NK**	**S**WM	4	20.0	42.5
HLA_DR2	1	143	FLG	VYYHKN**NKS**	WME	4	22.9	45.8
HLA_DR4oDRB1s0402c	2	143	FLG	VYYHKN**NKS**	WME	4	17.2	38.7
HLA_DR5	2	143	FLG	VYYHKN**NKS**	WME	4	20.4	42.0
HLA_DR1	1	144	LGV	YYHKN**NKS**W	MES	4	19.7	41.8
HLA_DR11oDRB1s1101c	19	144	LGV	YYHKN**NKS**W	MES	4	9.7	14.7
HLA_DR2	8	144	LGV	YYHKN**NK**SW	MES	4	14.4	28.8
HLA_DR11oDRB1s1101c	2	145	GVY	YHKN**NKS**WM	ESE	4	24.7	37.6
HLA_DR4	18	145	GVY	YHKN**NKS**WM	ESE	4	10.9	26.3
HLA_DR4oDRB1s0401c	4	145	GVY	YHKN**NKS**WM	ESE	4	17.1	38.9
HLA_DR7oDRB1s0701c	2	145	GVY	YHKN**NKS**WM	ESE	4	19.8	38.6
HLA_DQ8oDQA1s0301xDQB1s0302c	2	148	YHK	N**NKS**WMESE	FRV	4	15.2	29.7
HLA_DR8oDRB1s0801c	4	157	ESE	FRVYSSAN**N**	**CT**F	5	15.1	32.0
HLA_DR5	20	159	EFR	VYSSAN**NCT**	FEY	5	10.5	21.7
HLA_DR7	1	159	EFR	VYSSAN**NCT**	FEY	5	22.4	43.5
HLA_DR1oDRB1s0101c	8	160	FRV	YSSAN**NCT**F	EYV	5	13.8	28.7
HLA_DR3	5	166	AN**N**	**CT**FEYVSQP	FLM	5	13.0	32.3
HLA_DR4oDRB1s0402c	13	231	DLP	IGI**NIT**RFQ	TLL	6	11.5	25.8
HLA_DR7	9	234	IGI	**NIT**RFQTLL	ALH	6	13.0	25.3
HLA_DR1oDRB1s0101c	20	235	GI**N**	**IT**RFQTLLA	LHR	6	9.9	20.6
HLA_DR4oDRB1s0401c	7	235	GI**N**	**IT**RFQTLLA	LHR	6	14.5	32.9
HLA_DR4oDRB1s0402c	11	235	GI**N**	**IT**RFQTLLA	LHR	6	11.7	26.3
HLA_DR4	12	277	TFL	LKYNE**NGT**I	TDA	7	11.9	28.8
HLA_DR5	15	278	FLL	KYNE**NGT**IT	DAV	7	11.3	23.3
HLA_DR1oDRB1s0101c	22	285	**NGT**	ITDAVDCAL	DPL	7	9.6	20.0

HLA_DR8oDRB1s0801c	11	325	PTE	SIVRFP**NIT**	NLC	8	13.1	27.9
HLA_DR15oDRB1s1501c	3	326	TES	IVRFP**NIT**N	LCP	8	15.9	38.1
HLA_DR4	1	326	TES	IVRFP**NIT**N	LCP	8	16.2	39.2
HLA_DR15oDRB1s1501c	22	335	ITN	LCPFGEVF**N**	**AT**R	9	10.6	25.4
HLA_DR15oDRB1s1501c	11	341	FGE	VF**NAT**RFAS	VYA	9	13.4	32.0
HLA_DQ7oDQB1s0301c	10	611	VAV	LYQDV**NCT**E	VPV	10	13.1	28.8
HLA_DR7oDRB1s0701c	3	612	AVL	YQDV**NCT**EV	PVA	10	16.2	31.6
HLA_DR8oDRB1s0801c	14	703	GAE	NSVAYS**NNS**	IAI	11	12.5	26.5
HLA_DR4oDRB1s0401c	18	718	PT**N**	**FT**ISVTTEI	LPV	12	10.1	22.9
HLA_DR5	12	792	YKT	PPIKDFGGF	**NFS**	13	11.7	24.1
HLA_DQ8oDQA1s0301xDQB1s0302c	3	794	TPP	IKDFGGF**NF**	**S**QI	13	15.0	29.3
HLA_DR4	11	797	IKD	FGGF**NFS**QI	LPD	13	12.1	29.3
HLA_DR15oDRB1s1501c	12	799	DFG	GF**NFS**QILP	DPS	13	13.1	31.4
HLA_DR2	5	1066	LHV	TYVPAQEK**N**	**FT**T	14	15.3	30.6
HLA_DR7	5	1066	LHV	TYVPAQEK**N**	**FT**T	14	17.2	33.4
HLA_DR4	7	1072	PAQ	EK**NFT**TAPA	ICH	14	12.9	31.2
HLA_DR4	2	1095	EGV	FVS**NGT**HWF	VTQ	15	13.4	32.4
HLA_DR4oDRB1s0401c	2	1095	EGV	FVS**NGT**HWF	VTQ	15	17.7	40.2
HLA_DR7oDRB1s0701c	5	1095	EGV	FVS**NGT**HWF	VTQ	15	15.0	29.2
HLA_DR2	20	1130	DVV	IGIV**NNT**VY	DPL	16	12.5	25.0
HLA_DR8oDRB1s0801c	15	1135	IV**N**	**NT**VYDPLQP	ELD	16	12.3	26.0
HLA_DR2	17	1154	ELD	KYFK**NHT**SP	DVD	17	12.7	25.4
HLA_DR5	5	1154	ELD	KYFK**NHT**SP	DVD	17	15.9	32.8
HLA_DR7	6	1154	ELD	KYFK**NHT**SP	DVD	17	16.8	32.7
HLA_DR4oDRB1s0401c	1	1155	LDK	YFK**NHT**SPD	VDL	17	20.1	45.6
HLA_DR51oDRB5s0101c	4	1155	LDK	YFK**NHT**SPD	VDL	17	16.8	42.6
HLA_DR7oDRB1s0701c	12	1155	LDK	YFK**NHT**SPD	VDL	17	12.1	23.5
HLA_DR4oDRB1s0405c	4	1176	**NAS**	VVNIQKEID	RLN	18	15.0	35.8
HLA_DQ8oDQA1s0301xDQB1s0302c	7	1187	DRL	NEVAKNL**NE**	**S**LI	19	12.8	25.0
HLA_DR8oDRB1s0801c	16	1187	DRL	NEVAKNL**NE**	**S**LI	19	11.1	23.5

[a]Output from the Rankpep server (imed.med.ucm.es/Tools/rankpep)
